# Spatial Distribution Balance Analysis of Hospitals in Wuhan

**DOI:** 10.3390/ijerph13100971

**Published:** 2016-09-30

**Authors:** Nai Yang, Shiyi Chen, Weilu Hu, Zhongheng Wu, Yi Chao

**Affiliations:** 1 Faculty of Information Engineering, China University of Geosciences, 388 Lumo Road, Wuhan 430074, China; unichsy@163.com (S.C.); 15172508548@163.com (W.H.); chaoyi@cug.edu.cn (Y.C.); 2 NavInfo Co., Ltd., F16-17, TowerA, Beijing Phoenix Place, A-5, Shuguang Xili, Chaoyang District, Beijing 100028, China; keykeywu@hotmail.com

**Keywords:** hospital, accessibility, spatial distribution, Huff model, multi-criteria evaluation

## Abstract

The spatial distribution pattern of hospitals in Wuhan indicates a core in the central urban areas and a sparse distribution in the suburbs, particularly at the center of suburbs. This study aims to improve the gravity and Huff models to analyze healthcare accessibility and resources. Results indicate that healthcare accessibility in central urban areas is better than in the suburbs, where it increasingly worsens for the suburbs. A shortage of healthcare resources is observed in large-scale and high-class hospitals in central urban areas, whereas the resources of some hospitals in the suburbs are redundant. This study proposes the multi-criteria evaluation (MCE) analysis model for the location assessment in constructing new hospitals, which can effectively ameliorate healthcare accessibility in suburban areas. This study presents implications for the planning of urban healthcare facilities.

## 1. Introduction

Numerous countries have created local environments to provide residents access to healthcare resources in response to the initiatives of the World Health Organization [1,2]. However, hospitals vary in type, specialty, scale, and location, and patients often cross town or city boundaries for a doctor or hospital services, which is an inevitable consequence of dispersed populations and medical services [2,3]. Hospital services are strongly consumption- and service-oriented, and they require improved spatial distribution research to maintain socially fair resource allocation, to eliminate spatial polarization, and to reduce spatial differentiation.

Relevant studies have mainly focused on a balanced spatial distribution of medical infrastructure relative to the population, hospital level and scale, and service area. The location quotient is used to measure the degree of spatial aggregation of public service facilities [4] and to evaluate the distribution balance between the public facilities and the population [5]. The balance of medical resource allocation can be indirectly analyzed via the population health distribution determined using the Lorenz curve, the Gini coefficient, and the difference index [6]. A public service facility dominance model based on the gravity model has been developed to describe the healthcare accessibility of an area [7]. Determined using the improved two-step floating catchment area method, this study also reflects the spatial accessibility to primary health care services in different types of rural areas [8,9], and shows in the accessibility scores that there is imbalance in the southwest of Montreal [10]. Accessibility has long been recognized as key to the development of spatial planning strategies [11]. Four categories have been classified as measures of spatial accessibility to healthcare to assess the spatial distribution balance: provider-to-population ratios, distance to nearest provider, average distance to a set of providers and gravitational models of provider influence [12,13,14]. Patients prefer to choose the best hospitals, and the best hospitals always locate in crowed areas. In time, healthcare accessibility varies greatly, which causes spatial heterogeneity for the high-quality hospitals [15]. So the new construction of hospitals should be planned considering population, distance, transportation, and other factors [3,14]. Increased research on medical services and resource allocation for hospital construction is necessary to reduce the costs of medical treatments and to maximize the scope of medical services [16].

This study uses the ArcGIS (Esri, Redlands, CA, USA) to analyze the spatial distribution characteristics of hospitals in Wuhan. This study also utilizes the improved gravity and Huff models to analyze resident healthcare accessibility and medical resource usage, respectively. New medical construction areas in suburbs are assessed for areas lacking medical resources and accessibility based on their distance from existing hospitals and proximity to the population.

## 2. Study Area and Data

Wuhan is the capital of the Hubei Province in the Yangtze River Basin in Central China. The study area was selected using the 2009 Wuhan administrative map from the Hubei Bureau of Surveying, Mapping, and Geoinformation website [17]. The study area includes seven central urban areas (i.e., Jiang’an, Jianghan, Qiaokou, Hanyang, Wuchang, Qingshan, and Hongshan Districts) and six suburban areas (i.e., Dongxihu, Hannan, Caidian, Jiangxia, Huangpi, and Xinzhou Districts. The medical service facilities in this study are based on the Wuhan medical infrastructures. According to different professional fields, the Chinese health care system includes the general hospital, specialized hospital, teaching hospital, and community clinic. A total of 256 hospitals in this paper were divided into general hospitals (providing general or major subject medical service), specialized hospitals (providing a specific disease, special treatment methods and other special medical services), and Chinese traditional medicine hospitals. Furthermore, these hospitals were further categorized as third-, second-, and first-class hospitals on the basis of the quality of medical service and hospital scale. In China, the higher the class is, the better the hospital. A few unrated institutions were assigned to first-class hospitals. The location data were based on the Baidu Map [18]. The number of sickbeds was obtained from official hospital websites. For hospitals without sickbed data, the average sickbed numbers as per the appropriate rank of the hospital were used. For example, the sickbed numbers of a first- and second-class hospitals are 30 and 50, respectively. Land use classification data, as well as the information from 165 population points were obtained from the International Geosphere-Biosphere Program and the Wuhan Statistical Yearbook (2013) [19], respectively. The population points mean the population of the sub-district in accordance to the yearbook, and the location of the point is the location of the sub-district office. The road network data were extracted from ArcGIS Online.

## 3. Methods

### 3.1. Spatial Distribution Analysis

**Figure 1:**
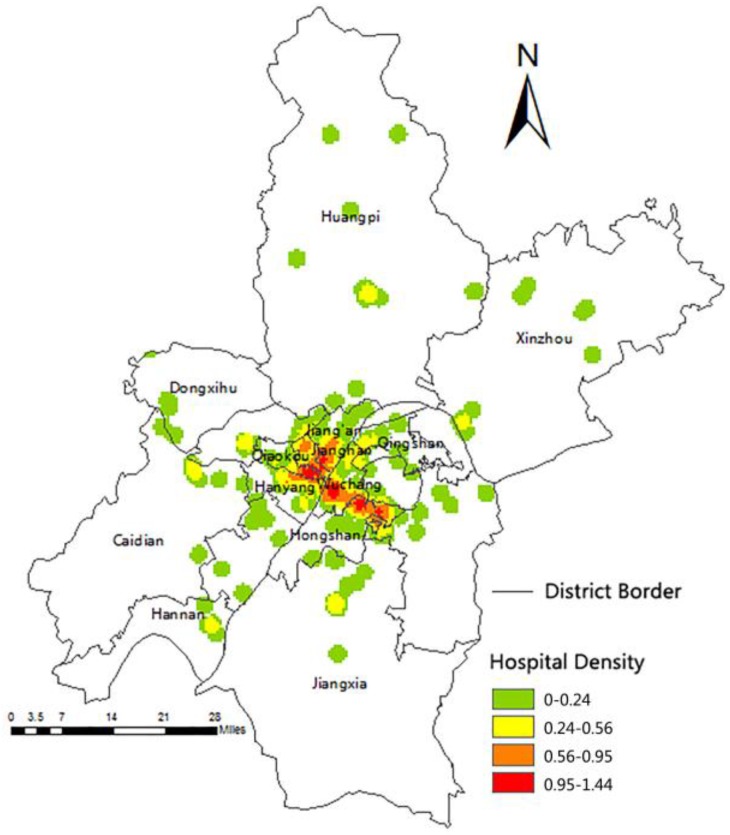
Wuhan hospital density.

**Figure 2:**
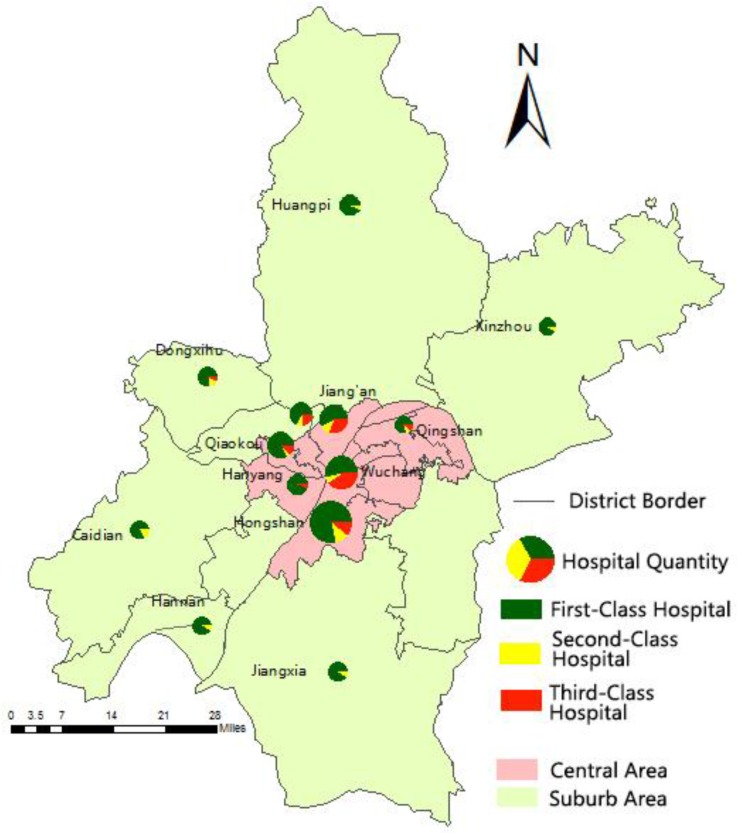
Wuhan hospital distribution.

The hospitals in Wuhan are mainly concentrated in the central urban area based on several experiments using point density analysis in ArcGIS within a 2000 scope (Figure 1). This scope of threshold is a parameter of this method which shows a better effect of hospitals aggregation. The densest area is spread along the river, with the hospital concentration, and gradually dispersed to the periphery. Healthcare service is intensive in central suburban areas. However, the supply in the central urban area is higher than in the suburbs (Figure 2). The hospitals are, overall, disproportionally distributed. Most areas do not have hospitals, such as the northern and the southwestern parts of the Hongshan District.

The spatial distribution density of hospitals in Wuhan is 0.03/km^2^ (Table 1). The hospital density in the central urban area is 0.23/km^2^. However, the density in the suburbs is below 0.01/km^2^. These findings prove that the healthcare coverage rate in the suburbs is lower than that in the central urban area. The core zone of the hospital distribution is located in the Jianghan and Qiaokou Districts. Third-class hospitals are mainly found in the Wuchang District, which accounts for 36.84% of all third-class hospitals in the Wuhan City (Figure 2). No third-class hospitals are found in the suburbs.

The comparison above shows numerous differences among the hospitals in these districts. The hospital quantity is imbalanced between the central urban area and the suburbs. From the perspective of the geographical distribution, the hospitals are widely distributed and have adequate supplies in the central area, whereas the distribution in the suburbs is sparse. Third-class hospitals are all in the central urban area. Moreover, the scale of hospital services is also imbalanced in the central urban area, which includes the Qingshan and Hanyang Districts, which hosts only one third-class hospital.

### 3.2. Spatial Accessibility Analysis

Health service planners require accurate, reliable, and robust measures to determine the spatial variation in the accessibility patterns and to ensure appropriate access to healthcare in the city [20]. Good and equal access to healthcare for the whole population, regardless of geography, remains a key goal of governments and societies [21]. The assessment of the spatial accessibility of medical services for people needs to consider the location, scale, and costs of peripheral hospitals.

#### 3.2.1. Improved Accessibility Analysis Model

In 1959, Hansen proposed the first law of geography as a measure of accessibility [22]. He considered all service facilities for the selection, which helped calculate the opportunity-accumulated value easily. $M_{j}/D_{ij}^{\beta }$ is the gravity force from *j* to *i*, where $M_{j}$ is the scale of *j*, $D_{ij}$ is the impedance between *i* and *j*, and β is the friction coefficient that describes travel impedance. The gravity model is expressed as following in Equation (1):

(1) $$A_{i}=\overset{n}{\underset{j=1}{\sum }}A_{ij}=\overset{n}{\underset{j=1}{\sum }}M_{j}/D_{ij}^{\beta }$$

This paper utilized the improved gravity model to calculate the accumulated value of gravitational force to evaluate the degree of healthcare accessibility. The equations are written as following in Equations (2) and (3):

(2) $$A_{i}=\overset{n}{\underset{j=1}{\sum }}\alpha_{j}S_{j}/V_{j}D_{ij}^{\beta }$$

and:

(3) $$V_{j}=\overset{m}{\underset{i=1}{\sum }}C_{i}/D_{ij}^{\beta }$$

where $A_{i}$ is the indicator of healthcare accessibility that represents population point *i* to all reachable hospitals, $\alpha_{j}$ is the class coefficient of the *j*-th hospital’s scale, $S_{j}$ represents the service ability, $V_{j}$ is the competition affecting population points around the *j*-th hospital and the limited medical resources, $C_{i}$ is the population quantity of the *i*-th population point, $D_{ij}$ is the time cost based on the actual transport network between the *i*-th population point and the *j*-th hospital, β is the impedance coefficient of distance, and *n* and *m* are the amounts of hospitals and population points, respectively.

The improved gravity model also considers the impact of population density on the healthcare accessibility indicator, which accounts for the influence of different-scale hospitals on the resident selection behavior. $S_{j}$ represents the hospital sickbeds and the service ability of the hospital, and $C_{i}$ is the population of this population point. The combination of the actual situation in Wuhan and the synthesis of the healthcare ability of each hospital shows that patients tend to choose professional hospitals for treatment. Therefore, $\alpha_{j}$ is 4 when the *j*-th hospital is a third-class specialized hospital, followed by 3, 2, and 1 for third-, second-, and first-class hospitals. β is the impedance indicator of distance. Scholars believe that β has different mathematical expressions (e.g., linear and exponential). The scale of β varies with the types of service and population group [13]. Considering other research methods on spatial accessibility [23], β was widely used when β=2 in this paper. The costs of the shortest commute between the population point and the hospital were selected as healthcare costs ($D_{ij}$).

#### 3.2.2. Realization of Improved Accessibility Analysis Model

The shortest time between each population point to the nearest hospital was determined as 0.644 h using the nearest facility analysis in ArcGIS. This value was set as the interrupt travel time of the OD matrix in network analysis in ArcGIS. All time costs and routes between the population points to all hospitals were then established within the interrupt value using the shortest transportation network route. The time value was used as the healthcare cost ($D_{ij}$) in the improved accessibility analysis model. The accessibility between the population point to the hospital is impossible when the routes exceed the interrupt travel time.

Equations (2) and (3) calculate the healthcare accessibility indicator of each population point, which is the convenience degree of resident medical treatment ($A_{i}$). The data are shown in Table 2.

**Figure 3:**
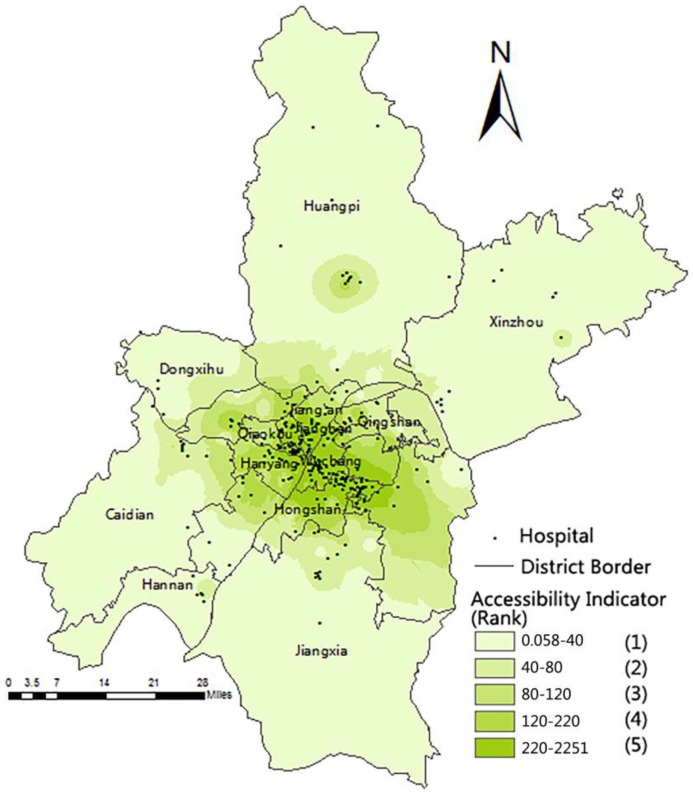
Medical accessibility of population points in Wuhan.

The data in Table 2 is hierarchically displayed on the map in Figure 2 using the Inverse Distance Weighted (IDW). This method assumes that the variable being mapped decreases in influence with distance from its measured location, which is to predict a value for any unmeasured location. Then the unknown accessibility power of anywhere will be estimated and displayed by IDW using the measured location values. Darker colors denote better healthcare accessibility. Figure 3 shows that most hospitals located in the central urban areas have better accessibility. Furthermore, places that are farther from the hospitals, which have a small accessibility indicator, are mainly in the suburbs (e.g., Anshan Street, Shu’an Town, and Mulan Street). On the whole, the accessibility was decreasing from the central urban areas to the suburbs. The places close to many hospitals have better accessibility, and they are mainly concentrated in the central city zone along the river, such as Liujiaoting and Huanghelou Street. The central urban area residents are provided with more medical resources, better resource quality, and shorter travel time than suburban residents.

#### 3.2.3. Different Accessibility Analysis

The reasons for the good accessibility of population points are as follows:

1. As the Equations (2) and (3) show, the population is negative for accessibility; thus, less population causes less negative effects.
2. One population point can reach multiple hospitals within the travel time limit.
3. Population points reach large-scale and high-class hospitals.

For instance, the Huanghelou Street is located in the central area of the Wuchang District from which 40 hospitals can be reached within the travel time limit. These hospitals offer good service, and they can be reached fast, thereby showing good accessibility. The reasons for poor accessibility are as follows:

1. The travel to hospitals takes long.
2. Few hospitals can be reached.

Shu’an Town is located in the south of the Jiangxia District at the border of Wuhan. The town is inaccessible and remote, with poor healthcare accessibility.

### 3.3. Treatment Analysis

#### 3.3.1. Improved Huff Model

Gravity modeling techniques significantly contribute to solving these retail network management issues [24]. The Huff model assumes that the consumption rate in a commercial facility is proportional to the effectiveness of the selection [25,26]. This solves the service area division problem for multiple stores and calculates the probability of selecting a commercial zone among consumers [27].

The Huff model can be appropriately used for the simulation of treatment situations of a population point and the frequency calculation of people seeing doctors in a hospital. The combination along with the research on the spatial distribution of medical facilities in Wuhan improves the Huff model.

(4) $$P_{ij}{=A}_{ij}/\overset{n}{\underset{j=1}{\sum }}A_{ij}$$

(5) $$E_{j}=\overset{n}{\underset{i=1}{\sum }}\left(P_{ij}C_{i}k\right)$$

(6) k=X/365Y

(7) $$H_{j}{=E}_{j}{/G}_{j}$$

$A_{ij}$ in Equation (4) is the gravitational force between the *j*-th hospital and the *i*-th population point, which relies on Equation (2). *P_ij_* is the medical probability between the *i*-th population point and the *j*-th hospital. In Equation (5), $E_{j}$ is the daily potential outpatient population; $C_{i}$ is the population of the *i*-th population point; and *k* is the coefficient of the daily average of outpatient population. In Equation (6), *X* is the number of outpatients in a whole year, and *Y* is Wuhan’s population in that year. In Equation (7), $H_{j}$ is the ratio of $E_{j}$ and $G_{j}$, where $G_{j}$ is the number of actual daily outpatients of the *j*-th hospital. The use of resources of the *j*-th hospital is reflected in the map by the $H_{j}$ value. If the ratio $H_{j}$ is less than 1, the actual demand of hospital resources is higher than the hospital supply. Therefore, this area lacks medical resources. Conversely, if the ratio is bigger than 1, the medical resources are redundant in that area.

#### 3.3.2. Realization of Improved Huff Model

The Wuhan Health Yearbook (2013) [28] shows that the total clinical patient service in 2012 in Wuhan equals 33,157,647 person-time, and the total population in Wuhan at the end of 2012 was 8,717,524. The coefficient *k* is 0.010421 based on the Equation (5). The daily potential outpatient populations of each hospital ($E_{j}$)—calculated using Equations (3), (4) and (6)—are shown in Table 3. 

Based on the year-round outpatients from National Most Hospital Directory and Outpatient report, the daily average of outpatients and their weighting in Wuhan hospitals were calculated and displayed in Table 4. This paper mainly studies general, Chinese traditional medicine, and specialized hospitals, which represent the most common types of hospitals that residents usually choose. The Wuhan Health Yearbook (2013) [28] indicated that the total outpatients ($Z_{r}$) in 2012 for the general, Chinese traditional medicine, and specialized hospitals (including maternal and child health hospitals) were 23,633,095; 2,985,144; and 4,252,292, respectively. According to the Equation (8), the actual daily average of outpatients (Table 5) for different classes and hospital types was calculated by weighting the values in Table 4.

(8) $$O_{rh}=Z_{r}W_{h}/365Q_{rh}$$

in Equation (8), *r* represents general, Chinese traditional medicine, and specialized hospitals; *h* represent third-, second-, first-class hospital; $O_{rh}$ is the actual daily average of outpatients; $Z_{r}$ is total outpatients about the *r*-th hospital, $W_{h}$ is the weighting of *h*-th hospital; $Q_{rh}$ is the sum of “*rh*” hospitals in Wuhan. For example, if *r* is for Chinese traditional medicine hospital and *h* is for third-class hospital, $Q_{rh}$ means the quantity of Chinese traditional medicine third-class hospitals.

The clinic ratio model was established using the daily potential outpatient quantity of each hospital from Table 3 and the daily actual average outpatients of each different class and different hospital type from Table 5 (see Equation (6) and Table 6).

**Figure 4:**
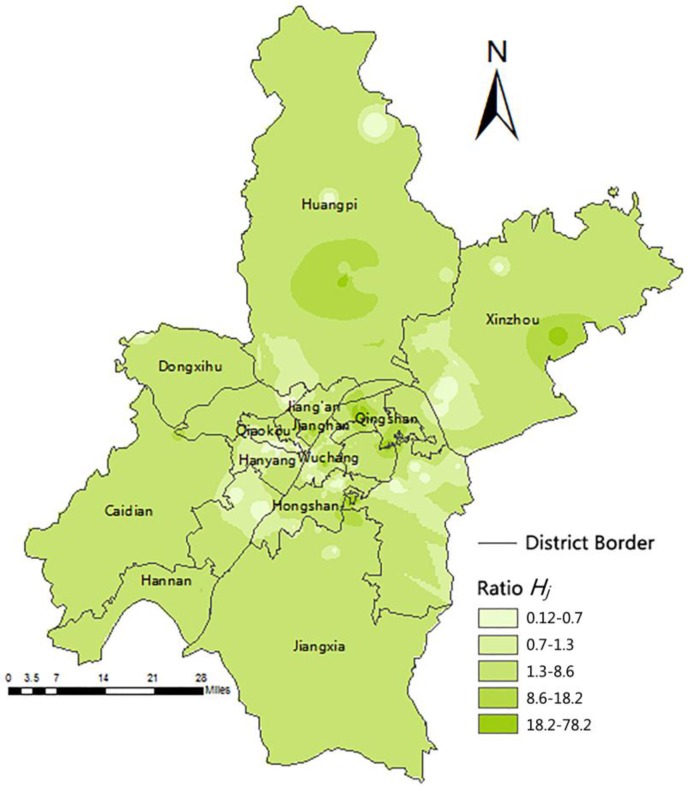
Ratio of potential daily outpatients and actual daily average of outpatients of each hospital in Wuhan in hypsometric layers.

Figure 4 shows that the data in Table 6 is reflected by the IDW in ArcGIS. The light-colored region represents the area with medical resource shortage, and the deep-colored region indicates areas with redundant resources. As a consequence from Figure 4, the most central urban areas are in shortage of medical resources, medical resources are adequate in the suburbs reversely, especially in the central district of suburbs.

#### 3.3.3. Difference Analysis of Outpatient Quantity

The actual number of outpatients was higher than the number of outpatients that existing medical resources can serve when the hospital ratio *H_j_* was less than 0.7. The light-colored area in Figure 3 has few medical resources, but on the other hand, some of them have high population densities, such as the northern part of the Hanyang District and the joint area between the Wuchang and Hongshan Districts, even though these areas have high-class, large-scale hospitals. However, the resources are limited. Hubei Maternal and Child Health Hospital in the Hongshan District serve beyond 100,000 people within 2 km. Moreover, residents from other distant places choose high-class hospitals around the central urban area for good medical service, which lead to resource shortage, because the actual outpatient quantity is higher than the outpatient capacity. Areas, such as the eastern part of the Hongshan District and the northern part of the Hannan District, have an adequate amount of medical resources when the ratio ranges between 0.7 and 1.3. The deep colored area with a ratio between 1.3 and 18.2 represents areas with minimal redundant resources. 

The resources of the suburbs are reasonable because the suburbs have few hospitals and few residents. A ratio (*H_j_*) above 1 reflects minimal redundancy for these large areas. Moreover, when the ratio is higher than 18.2, the medical resources in the area are redundant, such as in the center of the Huangpi and Xinzhou Districts. Combined with Section 3.1, despite there being better accessibility in central urban areas, there is a medical resources shortage here; meanwhile, there is adequate medical resources for few people with bad accessibility in suburbs.

Therefore, some effective measures should be taken to improve this phenomenon. The new hospital location should be selected according to different spatial accessibilities to solve medical resource shortage problems.

### 3.4. Hospital Location Selection Based on the Multi-Criteria Evaluation Analysis

The multi-criteria evaluation (MCE) analysis provides a solution for program assessment that includes multiple factors, multiple standards, and conflicting factors [14]. Combined with the geographic information system, thematic maps can be generated to indicate risk degrees [29]. New hospital locations were found by MCE in regions with resource shortages and bad accessibility.

#### 3.4.1. MCE Model

Based on a review in the Urban Public Facilities Planning Specification (GB50442-2008) and research on the maximum usage of limited data, the criteria for hospital location selection and the layout in Wuhan are as follows (Table 7). The larger the weight is, the more important the criterion is.

1. Transportation around the location is convenient.
2. The hospital is close to a densely populated area.
3. The new location is far from existing hospitals.
4. The area and scale of the new hospital should be appropriate.

The hierarchical data for rule A were reclassified according to the legend in Figure 3, and the results are presented in the brackets named Ranks from 1 to 5. The smaller the rank number is, the lower the accessibility is, which means that more new hospitals are necessary.

**Figure 5:**
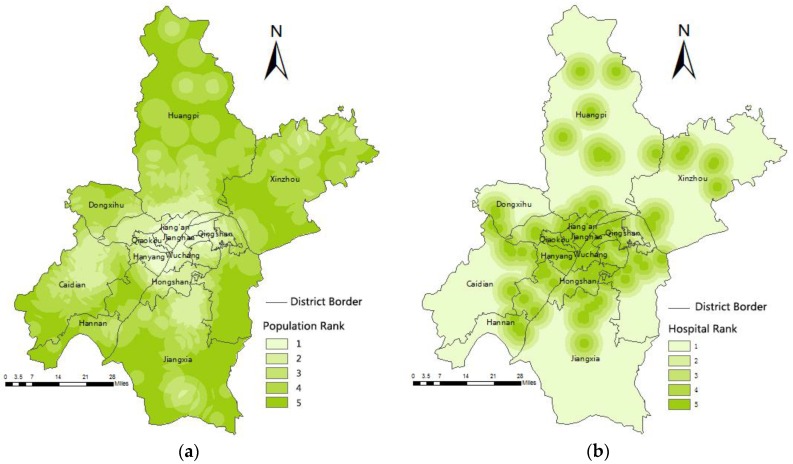
Suitable map after reclassification. (**a**) These regions refer to the population points. The lower the value is, thegreater the population intensity is. (**b**) These regions are about existing hospitals. The lower the value is, the more a new hospital is necessary.

The medical resource competition between different population points in rule B is reflected by reclassifying the population points according to the population quantity and the weighting of results (Table 8). The population suitable map was obtained using the Euclidean distance. The Euclidean distance output raster contains the measured distance from every cell to the nearest source. After this analysis, the map was reclassified afterwards. Ranks 1 to 5 represent the subsections of 0–2, 2–4, 4–6, 6–8, and >8 km. The final result *PG* means the density of people, which was calculated using the weight in Table 8 and Equation (9), as shown in Figure 5a. A lower value indicates a larger population density in the area.

After reclassifying the population data, the dataset was calculated by Equation (9).

*PG* = *a* × 0.3 + *b* × 0.2 + *c* × 0.2 + *d* × 0.2 + *e* × 0.1 (9)

for rule C, the hospitals were analyzed using the Euclidean distance, and the raster results were reclassified. The reclassification standards were the same as those used for rule B. The results are shown in Figure 5b. A lower value indicates smaller hospitals in this area.

For rule D, the reclassification and ranking of the legend were performed according to the same standards in Figure 4. 

#### 3.4.2. MCE Model Realization

**Figure 6:**
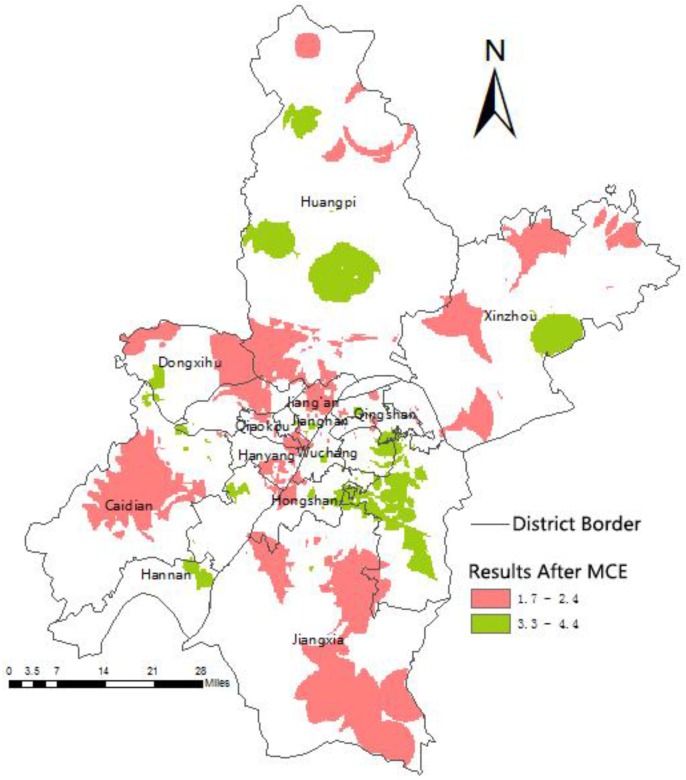
Unremoved non-building land location selection results. MCE = multi-criteria evaluation.

**Figure 7:**
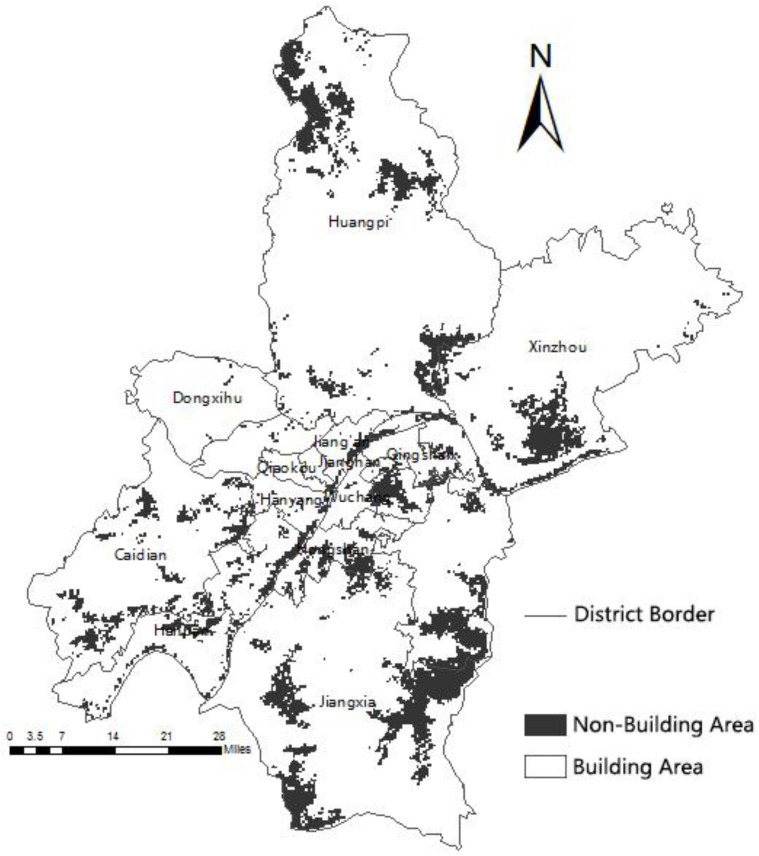
Land use classification map.

**Figure 8:**
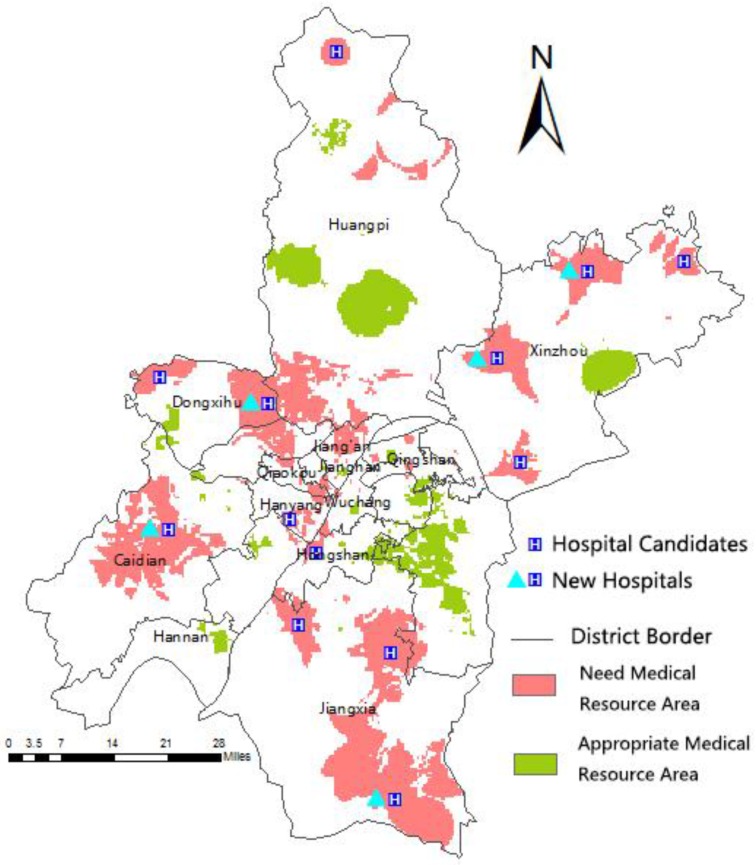
Removal of the non-building land location selection results.

According to the criteria and weights, the equation for the hospital location selection can be expressed as following in Equation (10):

*SUM* = *A* × 0.3 + *B* × 0.3 + *C* × 0.1 + *D* × 0.3 (10)

the Equation (10) findings and the ArcGIS Raster Calculator (Figure 6) calculation results were overlaid with the land use classification raster map (Figure 7) resulting in Figure 8. A lower value reflects a higher urgency for medical resources or hospitals. The red colored area represents these urgent areas, whereas the green colored areas denote those with appropriate resources.

#### 3.4.3. MCE Model Assessment

The red region in the central urban area shows insufficient per capita healthcare resources because of the large population density, even though the accessibility is good. Thus, the existing hospitals should be expanded or the doctor number should be increased in the red regions to ease the pressure of a large population density and to ameliorate the availability of medical resources. On the other hand, the red regions in the suburbs are areas without hospitals and with bad accessibility; whereas several population points need healthcare here and these suburban areas have sufficient medical resources. Thus, transforming existing hospitals into higher-class hospitals and building additional first-class hospitals or health centers in remote areas would be beneficial.

The centers in the red region were regarded as candidates for new suburban hospital locations for verifying the feasibility of the location selection method (Figure 8). The new hospital location was used as the facility point, whereas the population point was used as the requesting point in the location-allocation model in ArcGIS. Several locations which needed minimum facilities for service and covered a maximum part of the land were selected. A large number of experiments supposed that first-class hospitals should be constructed in the suburbs with an average of 30 sickbeds. Thus, the medical accessibility for residents in the suburbs can be improved by building only seven hospitals.

No notable effects on the central urban area were observed after new hospital locations were selected. However, accessibility in the suburbs was affected (Table 9). Only the Hangyang District showed increased medical accessibility among the seven central districts. The Xinzhou District displayed a larger rate of suburban area increase, whereas the Dongxihu District indicated a rate lower than those of the other districts. The overall medical accessibility indicators increased by 0.163%. The accessibility in the central urban and suburban areas increased by 0.085% and 2.682%, respectively.

## 4. Conclusions

This research demonstrated the following:

1. The majority—71.5%—of the hospitals are in the central urban area in Wuhan. The hospital density of Wuhan reached 0.23 places/km^2^, whereas, it was less than 0.01 places/km^2^ in the suburbs.
2. The medical accessibility in the central urban area is better than in the suburbs. Regions with good accessibility are located in the central area. Accessibility decreases from the central area to the suburbs.
3. Although there is better healthcare accessibility, medical resources are short for people in the central urban area. The suburbs have bad healthcare accessibility but there is sufficent medical resources.

Combined with land use classification information and MCE analysis, healthcare accessibility was improved. Seven new hospital locations were chosen by the ArcGIS location allocation model to ameliorate accessibility in the suburbs. 

With the methods of the improved gravity model and Huff model, the distribution characteristics of hospital in Wuhan could be understood, as well the regional accessibility and usage of medical resources as well. Then we put forward some solutions to improve the medical services for these factors. While more factors should be considered in this research. More types of hospitals should be considered for this research, such as community clinics. The location selection of some specialized hospitals that only for women or men or some age grades should be considered with sex or age standardized, rather than the whole population. Large-scale population data were used for this research because of the lack of population data on housing estates. Whether urban residents at the city border would select hospitals in the Wuhan city remains unclear. The relevant factors that influenced the research results of this paper should be further refined in a future study.

## Figures and Tables

**Table 1:** Wuhan hospital information.

District	Property	Population (Thousand)	Acreage (km^2^)	Population Density (person/km^2^)	Hospital	Hospital (%)	Density of the Hospital (place/km^2^)
Jianghan	Central	713.10	33.43	21,331	18	7	0.538
Qiaokou	Central	848.30	46.39	18,286	23	9	0.496
Jiang’an	Central	926.80	64.24	14,427	26	10.2	0.405
Wuchang	Central	1246.80	87.42	14,262	35	13.6	0.400
Qingshan	Central	51.26	68.40	7494	10	4	0.146
Hangyang	Central	61.67	108.34	5692	14	5.5	0.129
Hongshan	Central	147.74	480.20	3077	57	22.3	0.119
Hannan	Suburb	12.68	287.70	441	12	4.6	0.042
Dongxihu	Suburb	50.06	439.19	1140	13	5.1	0.030
Caidian	Suburb	66.34	1108.10	599	11	4.3	0.009927
Xinzhou	Suburb	85.57	1500.00	570	10	4	0.006667
Huangpi	Suburb	89.78	2261.00	397	15	5.8	0.006634
Jiangxia	Suburb	83.40	2010.00	415	12	4.6	0.005970
Sum	-	1022.00	8494.41	-	256	100	0.030137

**Table 2:** Healthcare accessibility indicator of each population point.

Population Point Name	$A_{i}$
Minzu Street	304.558
Minquan Street	424.503
…	…
Anshan Street	4.256
Liujiaoting	1964.135
Wangjiadun Airport	298.273
Jianghan Economic Development Zone	111.175

$A_{i}$ is the indicator of healthcare accessibility that represents population point *i* to all reachable hospitals.

**Table 3:** Daily potential outpatient population of each hospital in Wuhan.

Hospital Name	Daily Potential Outpatient Population $E_{j}$ (Person-Time/Hospital)
The Hospital of South-central University for Nationalities	15
Dongshan Hospital	22
Hubei General Hospital	2572
…	…
Jiangxia Traditional Chinese Medicine Hospital	64
Chinese People’s Liberation Army 161st Central Hospital	1134

**Table 4:** Daily outpatient population and weighting in Wuhan.

Hospital Class	Third-Class Hospital	Second-Class Hospital	First-Class Hospital	Sum
Daily Average Outpatients (Person-Time)	2300	250	150	2700
Weighting of Outpatient ($W_{h}$)	0.85	0.09	0.06	1

**Table 5:** Daily actual average of outpatients in Wuhan.

Hospital Class	Hospital Type	Daily Actual Average Outpatients $O_{rh}$ (Person-Time/Hospital)
Third-Class Hospital	General Hospital	2201
	Chinese Traditional Medicine Hospital	2317
	Specialized Hospital	1142
Second-Class Hospital	General Hospital	243
	Chinese Traditional Medicine Hospital	245
	Specialized Hospital	736
First-Class Hospital	General Hospital	27
	Chinese Traditional Medicine Hospital	29
	Specialized Hospital	21

**Table 6:** Ratio of potential daily outpatients and actual daily average of outpatients for each hospital in Wuhan (Portion).

Hospital Name	Ratio
Hospital of the South-Central University For Nationalities	0.56587
Dongshan Hospital	0.820014
Hubei General Hospital	1.168633
…	…
Jiangxia Traditional Chinese Medicine Hospital	2.361732
Chinese People’s Liberation Army 161st Central Hospital	0.51528

**Table 7:** Criteria and normalized weight allocation of location selection.

Criteria	Normalized Weight
Area of Bad Accessibility A	0.3
Close to the Population Point B	0.3
Far from the Existing Hospital C	0.1
Lack of Resources Area D	0.3
The Scale of the Hospital Matches the Population	Later to Judge

**Table 8:** Population-normalized weight allocation.

Type of Population	Weight
>40,000: a	0.3
30,000–40,000: b	0.2
20,000–30,000: c	0.2
10,000–20,000: d	0.2
0–10,000: e	0.1

**Table 9:** Changes after the addition of new hospitals.

District	Property	Number of New Hospitals	Average of Accessibility Indicator for Population Point before Adding Hospital	Average of Accessibility Indicator for Population Point after Adding Hospitals	Increase (%)	Central/Suburb Area Increase (%)
Jianghan	Central	0	400.331	400.483	0.038	0.085
Qiaokou	Central	0	516.093	516.297	0.04	
Jiang’an	Central	0	328.065	328.197	0.04	
Wuchang	Central	0	580.231	580.306	0.013	
Qingshan	Central	0	102.124	102.165	0.039	
Hangyang	Central	1	172.565	173.372	0.468	
Hongshan	Central	0	186.402	186.324	−0.042	
Hannan	Suburb	0	14.907	15.003	0.646	2.682
Dongxihu	Suburb	1	61.443	61.714	0.442	
Caidian	Suburb	1	18.543	19.397	4.604	
Xinzhou	Suburb	2	9.159	9.602	4.842	
Huangpi	Suburb	1	18.973	19.383	2.16	
Jiangxia	Suburb	1	15.494	16.021	3.402	
Sum	-	7	191.732	192.045	0.163	-
